# Strengthening national public health institutes: a systematic review on institution building in the public sector

**DOI:** 10.3389/fpubh.2023.1146655

**Published:** 2023-05-18

**Authors:** Lucia Brugnara, Catalina Jaramillo, Margarita Olarte-Peña, Larissa Karl, Andreas Deckert, Michael Marx, Olaf Horstick, Peter Dambach, Angela Fehr

**Affiliations:** ^1^evaplan GmbH at the University Hospital Heidelberg, Heidelberg, Germany; ^2^Heidelberg Institute of Global Health, Faculty of Medicine, University of Heidelberg, Heidelberg, Germany; ^3^Faculty of Natural and Social Sciences, Heidelberg University of Education, Heidelberg, Germany; ^4^Centre for International Health Protection, Robert Koch Institute, Berlin, Germany

**Keywords:** institution building, organization development, organization strengthening, institutional capacity building, national public health institute, international cooperation, public health organization

## Abstract

**Introduction:**

Strong and efficient institutions are vital to the development of well-functioning governments and strong societies. The term “institution building” encompasses the creation, support, development, and strengthening of organizations and institutions. Still, there is little aggregated evidence on “institution building” considering a wider system-thinking approach, best practices, or development cooperation specifically in the field of public health. In 2007, the International Association of National Public Health Institutes (IANPHI) created a guiding Framework that countries may use for developing National Public Health Institutes (NPHIs). This Framework is currently being revised.

**Methods:**

In this context, we conducted a systematic review to facilitate this revision with recent evidence on institution building and its potential contribution to NPHI. We followed the PRISMA guidelines for systematic reviews, searching for relevant publications in seven scientific databases (Pubmed, VHL/LILACS, EconLit, Google Scholar, Web of Science, World Affairs Online, ECONBIZ) and four libraries (World Bank; European Health for All database of the World Health Organization European Region, WHO; Organization for Economic Cooperation and Development, OECD; and the African Union Common Repository). The search was carried out in October 2021. We used the “framework analysis” tool for systematically processing documents according to key themes.

**Results:**

As a result, we identified 3,015 records, of which we included 62 documents in the final review. This systematic review fills a major gap of aggregated information on institution building in the field of public health and National Public Health Institutes. It is to our knowledge the first systematic review of this kind. The overriding result is the identification and definition of six domains of institution building in the health sector: “governance,” “knowledge and innovation,” “inter-institutional cooperation,” “monitoring and control,” “participation,” and “sustainability and context-specific adaptability.”

**Discussion:**

Our results show that the described domains are highly relevant to the public health sector, and that managers and the scientific community recognize their importance. Still, they are often not applied consistently when creating or developing NPHIs. We conclude that organizations engaged in institution building of NPHIs, including IANPHI, may greatly benefit from state-of-the-art research on institution building as presented in this study.

## 1. Introduction

The United Nations recognize the importance of solid institutions by establishing the aim of “building effective, accountable, and inclusive institutions at all levels” as a central part of the 16th Sustainable Development Goal (1, 2). The concept of “institution” is broad and not uniformly defined, ranging from “humanly devised constraints that structure political, economic, and social interactions” (3, 4) to a mere synonym for organization (5). Still, institutions can be built and strengthened by design (6, 7). The processes aiming to create, support, or develop institutions can be summarized under the term “institution building” (8).

Institution building initiatives and processes have been implemented in different contexts and sectors (9–16). For the specific case of the public health sector, National Public Health Institutes (NPHIs) are established to provide “science-based leadership, expertise, and coordination of a country’s public health activities,” among other core functions (17). Policy-setting and suitable public health decision-making should be outlined based on scientific knowledge, data, analysis, and evidence (18). While NPHIs shall be developed along core functions, based on essential public health functions, and encompass core attributes, their actual scopes of work and governance structures differ between countries (17, 19). Also, their mandates may be challenged because of the “schism” between the cultures of “medicine” and “public health” (20).

Organizations such as the International Association of National Public Health Institutes (IANPHI) or the World Health Organization (WHO), as well as foundations and financing agencies, supported the development and strengthening of NPHIs over the last decades (21). They provided technical assistance, capacity-building support, and funding, thereby promoting governance and institution building in public health globally. IANPHI, founded in 2006, or the Africa Centres for Disease Control and Prevention (Africa CDC) support the creation and development of NPHIs and, for this purpose, created frameworks and guidelines (17, 22). These guidelines describe the core functions of NPHIs, including essential public health functions, core attributes, and processes for their creation and enhancement. Strong leadership, clear identification of the organizational functions, development of strategic plans, and the importance of country ownership are vital aspects to be considered for NPHI institution building and continuous strengthening processes. Health experts and managers are aware of the importance of such components (23).

Aggregated evidence on successful “institution building” considering the system-thinking approach in the field of public health is scarce, as publications usually describe or analyze only specific components of institution building. A system-thinking approach that aims to look at issues as a larger and interconnected system could help to identify and address complex and interdependent factors that may influence institution building, as already demonstrated in other areas in the health sector (24, 25).

In the context of its new Strategy 2021–2025 and the Action Plan for implementation, IANPHI is revising its “Framework for the Creation and Development of NPHIs” (17).The IANPHI Executive Board has tasked the Robert Koch Institute and the Norwegian Institute of Public Health to lead the process of the Framework’s revision. These organizations seek to base this process on a comprehensive overview of the available evidence of institution building in the field of public health.

The objective of this study is to critically review the available literature and synthesize the evidence regarding public institution building, with a specific focus on public health and national public health institutes. This systematic review provides elements to the general audience working on the creation and development of public health institutions, it also supports and facilitates relevant processes carried out by IANPHI. We provide an overview of state-of-the-art methodological approaches, guiding documents, and evaluations regarding institution building in the public sector and the health sector, identifying best practices or guidance related to institution building in general, institution building as part of development cooperation, and institution building in NPHIs, specifically.

## 2. Materials and methods

### 2.1. Search strategy and eligibility criteria

The systematic review followed the PRISMA guidelines and checklist for reporting the results (26). We searched in seven scientific databases: Pubmed, VHL/LILACS, EconLit, Google Scholar, Web of Science, World Affairs Online, and ECONBIZ. Additionally, we searched in four virtual libraries of international organizations: World Bank, European Health for all Database (under the World Health Organization, WHO, European Region), Organization for Economic Cooperation and Development (OECD), and the common repository of the African Union. The search was carried out in October 2021. The search string combined term variations of three main categories: institution building, the outcome of interest (theories and concepts, framework and guidelines, empirical evidence of case reports, evaluations, studies), and public institutions. We received support from a librarian at the University of Heidelberg to create the final search strategy. The full search strategy is described in Supplementary material and our study protocol. The protocol was registered in PROSPERO in August of 2021 under the number CRD42021273702.

We included documents based on their contents (documents describing theories, policies, frameworks, guidelines, or evaluations of institution building in the public sector at regional, national or subnational levels, focusing on the public health sector) and records published from 2006 onwards since the IANPHI foundation, as we consider that the current IANPHI NPHI Framework, published in 2007 (17), was based on the relevant bibliography available at that time. We excluded documents with a focus on the private sector, specific public sector themes that were not transferable to public health, documents describing only legal frameworks, literature covering information at local or community level initiatives, and documents that did not clearly suggest or describe *how* to tackle challenges to build to strengthen public institutions. We also excluded documents exclusively considering financial aspects of institution building. We did not apply any language restrictions.

### 2.2. Data collection, quality appraisal, and data analysis

We used the electronic reference manager Mendeley for managing the citations and the electronic tool Covidence for the title and abstract screening, the full-text review, and the data extraction stages. We used Microsoft Excel to elaborate tables for quality assessment and data analysis. Two independent reviewers performed the screening, review, data extraction, and quality assessment. Discrepancies between the two reviewers were solved via discussions, and, when necessary, including the opinion of a third reviewer. Duplicates were removed before the title and abstract screening and full-text review stages in Covidence.

We used and adapted several tools for the quality appraisal of the documents: (1) Mixed Methods Appraisal Tool (MMAT) for peer-reviewed articles containing qualitative methods, mixed-methods, and quantitative descriptive methods (27), and (2) quality assessment of systematic reviews and meta-analyses NIH-NHLBI (28). We applied the AACODS (Accuracy, Authority, Coverage, Objectivity, Date, Significance) checklist (29) for the documents that did not match the criteria of the first two methods. This checklist was used for the documents classified as gray literature, and peer-reviewed documents that were non-systematic reviews, used only qualitative methods, or did not state the methodology used. Each of the tools had specific questions based on which the documents were classified. The lists of questions for each tool are described in the Supplementary material. We assigned the following scores for each one of the questions in the tools accordingly: (a) “Yes” responses were scored as “1”; (b) “No” responses were scored as “0”; (c) “Not Applicable” was not scored and it was not considered in the final score weight. We calculated a mean with an equal weight percentage for all questions and categories to get the final score. Only for the AACODS checklist, we assigned double weight for the “authority” and “significance” categories as we considered them the most relevant aspects for institution building, as, respectively, these criteria inform about the expertise of the organizations and authors working in the related fields (in our case, institution building), and if the elements of research or experience described in the document could be replicated. We included in the review all the documents that complied with the eligibility criteria, independently of their quality appraisal scores. For the data collection, we extracted general document information (e.g., author, year of publication, main affiliation/donor, study design), purpose/aim of the document, and our outcomes of interest (theories and concepts on institution building, frameworks and guidelines on institution building, and empirical evidence of initiatives on institution building), and main findings based on the six identified institution building domains. To identify the main domains in institution building, we used the “framework analysis” (30), a qualitative data analysis method for policy research and systematically processing documents according to key themes. With this method, we first assessed all documents and identified common domains described in more than one publication. Afterwards, we reexamined all documents, extracted and analyzed the data, and reported the results based on the domains identified.

## 3. Results

**Figure 1:**
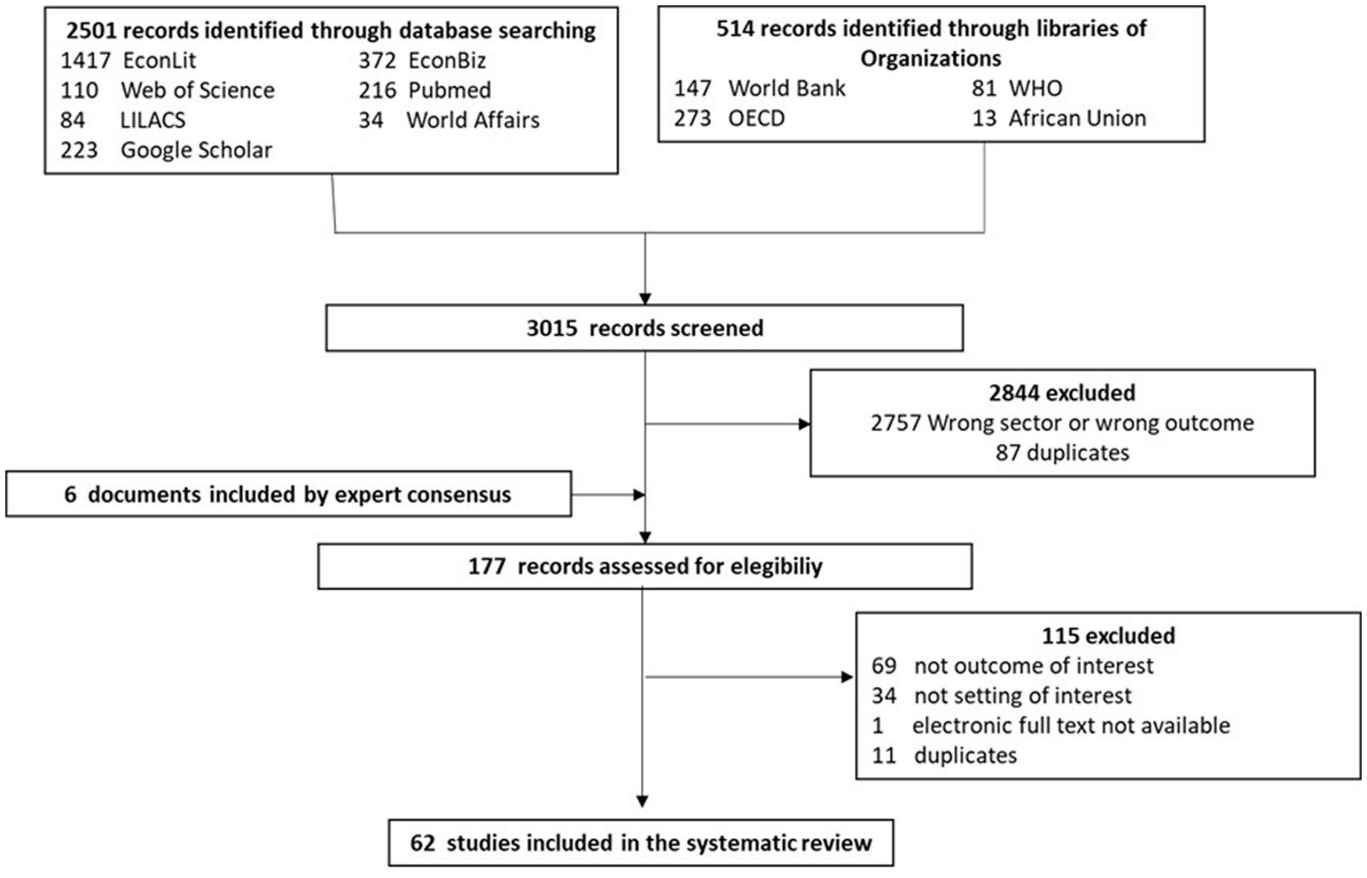
PRISMA flow-chart of the study selection process.

The electronic search resulted in 3015 records, of which 87 duplicates were removed. We screened 2,928 records by title and abstract, retrieving 171 records for full-text review. We added six background references that were not found in our systematic search but, by expert consensus, were included before the full-text review stage. Of the 177 records assessed for eligibility, we excluded 115: 69 did not describe our outcomes of interest, 34 did not describe our setting or our interest (e.g., publications about the private sector), one electronic text was not available, and 11 documents were duplicates. Finally, we selected 62 records for data extraction and analysis (Figure 1).

### 3.1. Characteristics of source of evidence

We classified the documents according to the following characteristics: year of publication, geographical origin of the reported information (Table 1), type of document (Table 2), and field/sector related to institution building (Table 3).

**Table 1:** Classification of sources of evidence according to the geographical origin of the reported information.

Region	#	References
Sub-Saharan Africa	17	African Union and Africa CDC (2019) (22); Alam et al. (2016) (31); Barma et al. (2014) (5); Barzilay et al. (2018) (32); Bridges and Woolcook (2017) (33); Clement et al. (2015) (34); De Bruyn 2019 (35); Erondu et al. (2021) (36); Ihekweazu et al. (2015) (37); Johnstone et al. (2019) (38); Khan et al. (2020) (39); Manoj et al. (2020) (40); Marjanovic et al. (2013) (41); Meda et al. (2016) (42); Rosenfeld et al. (2020) (43); World Bank and Government of Rwanda (2020) (44); Zaato and Ohemeng (2015) (45)
Europe and Central Asia	12	Aluttis et al. (2014) (46); Desai and Snavely (2007) (47); Eriksen et al. (2007) (48); Ihekweazu et al. (2015) (37); Lenz (2021) (49); Leovaridis and Popescu (2015) (50); Lopert et al. (2017) (51); Pelling et al. (2008) (52); Radin (2020) (9); Ruseva et al. (2015) (53); Salkic (2014) (54); Taytak and Aydin (2020) (55)
North America	3	Boyd (2011) (56); Gagnon and Seguin (2010) (57); Mayer et al. 2019 (58)
South Asia	4	Erondu et al. (2021) (36); Khan et al. (2020) (39); Lejars (2008) (59); Rao and Kandelwal (2016) (60)
East Asia and Pacific	4	Bloom (2012) (61); Essink et al. (2020) (62); Hudalah et al. (2014) (63); Song (2009) (64)
Latin America and the Caribbean	3	Barzilay et al. (2018) (32); González-Block (2009) (65); Portes and Smith (2010) (10)
Middle East and North Africa	1	Abdellatif et al. (2019) (66)

**Table 2:** Characteristics of sources of evidence according to type of document.

Outcome (type of information)	#	Type of document	#	Study design	#	References
Concepts review and theories	16	Gray literature	5	Descriptive analysis/Policy paper	5	OECD (2014) (67); Rao and Khandelwal (2016) (60); Reinold (2017) (68); Treviño (2016) (69); World Bank and Government of Rwanda (2020) (44)
		Peer-reviewed article	11	Descriptive analysis	11	Desai and Snavely (2007) (47); Edelstein et al. (2018) (70); Eriksen (2007) (48); Khan et al. (2020) (39); Jakab et al. (2021) (14); Lejano (2006) (71); Leovaridis and Popescu (2015) (50); Mahoui and Ferfera (2013) (72); Naimoli and Saxena (2018) (73); Verrecchia et al. (2019) (74)
Frameworks and guidelines	21	Gray literature	5	Descriptive analysis/ Policy paper	5	African UnionAfrica CDC (2019) (22); Cole and McGinnis (2017) (75); Lopert et al. (2017) (51); OECD (2020a) (76); Savedoff (2011) (77); World Bank (2014) (78)
		Peer-reviewed	16	Qualitative study	1	Zaato and Ohemeng (2015) (45)
				Mixed-methods study	2	Abdullahi et al. (2016) (79); Strielkowski et al. (2020) (80)
				Essay	2	Munteanu and Newcomer (2020) (81); Romanelli (2017) (82)
				Systematic review	1	Manoj et al. (2020) (40)
				Descriptive/ Policy analysis	10	Abdellatif et al. (2019) (66); Aluttis et al. (2014) (46); Bloom and Wolcott (2013) (83); Buntaine et al. (2017) (84); Clement et al. (2015) (34); Meda et al. (2016) (42); Pelling et al. (2008) (52); Rosenfeld et al. (2020) (43); Ruseva et al. (2015) (53); Taytak and Aydin (2020) (55)
Empirical evidence	24	Gray literature	5	Descriptive analysis, report	5	Alam et al. (2016) (31); Barma et al. (2014) (5); Bridges and Woolcock (2019) (33); OECD (2020b) (85); Radin (2020) (9)
		Peer-reviewed	19	Mixed-methods study	1	Essink et al. (2020) (62)
				Qualitative study	1	Mayer et al. (2019) (58)
				Descriptive/ Policy analysis/Case study	13	Barzilay et al. (2018) (32); Bloom (2011) (61); De Bruyn (2019) (35); Erondu et al. (2021) (36); Gagnon and Seguin (2010) (57); González-Block (2009) (65); Hudalah et al. (2017) (63); Ihekweazu et al. (2015) (37); Johnstone et al. (2019) (38); Lejars (2008) (59); Lenz (2021) (49); Marjanovic et al. (2013) (41); Song (2009) (64)
				Cross-sectional study	3	Boyd (2011) (56); Portes and Smith (2019) (10); Salkic (2014) (54)
				Commentary	1	Binder et al. (2008) (23)
Other	1	Peer reviewed	1	Modelling study	1	Grajzl and Murrell (2009) (86)

**Table 3:** Characteristics of sources of evidence according to field or sector related to institution building.

Field/sector of Institution building	#	References
Health/Public Health	22	African Union and Africa CDC (2019) (22); Aluttis et al. (2014) (46); Barzilay et al. (2018) (32); Binder et al. (2008) (23); Bloom (2011) (61); Bloom and Wolcott (2013) (83); De Bruyn (2019) (35); Edelstein et al. (2018) (70); Erondu et al. (2021) (36); Ihekweazu et al. (2015) (37); Jakab et al. (2021) (14); Johnstone et al. (2019) (38); Khan et al. (2020) (39); Lejano (2008) (71); Lejars (2008) (59); Manoj et al. (2020) (40); Mayer et al. (2019) (58); Naimoli and Saxena (2018) (73); Portes and Smith (2010) (10); Rosenfeld et al. (2020) (43); Savedoff (2011) (77); Verrechia et al. (2019) (74)
Education	2	Desai and Snavely (2007) (47); Strielkowski et al. (2020) (80)
Defense	1	Radin (2020) (9)
Water	1	Zaato and Ohemeng (2015) (45)
Environment	1	Clement et al. (2015) (34)
Not stated/Public Administration and/or institution building in general	35	

Among the 62 selected records, 17 were published between 2006 and 2013. The remaining 45 documents were published between 2014 and 2021. We further classified documents according to geographic regions or country of the study, initiative, or reporting. For documents describing cooperation between organizations or countries in more than one region, we differentiated as follows: if activities and support received were prominent in one specific country, we considered the respective country; if collaboration and activities were balanced between countries, we considered both countries. Following this, 17 sources of evidence reported information related to Sub-Saharan Africa, 12 included information from Europe and Central Asia, three records reported data from North America, four from South Asia, four from East Asia and Pacific, three from Latin America and the Caribbean, and one from the Middle East and North Africa. In addition, 21 sources of evidence that were analyzed included general information without stating a specific country or region.

### 3.2. Quality appraisal of documents according to the characteristics of the information and study design

Table 4 presents the final scoring of the quality of the documents included in this systematic review. Most of the documents had a very high score based on the criteria used; only eight documents scored below 0.8.

**Table 4:** Quality appraisal scores.

	Tool	Author	Peer reviewed/Gray literature	Score
1	AACODS	Abdellatif et al., 2019 (66)	Peer reviewed	0.86
2		African Union/Africa CDC, 2019 (22)	Gray literature	0.51
3		Alam et al., 2016 (31)	Gray literature	0.88
4		Aluttis et al. (46)	Peer reviewed	0.88
5		Barma et al., 2014 (5)	Gray literature	0.86
6		Barzilay et al., 2018 (32)	Peer reviewed	0.88
7		Binder et al., 2008 (23)	Peer reviewed	0.88
8		Bloom and Wolcott, 2013 (83)	Peer reviewed	0.84
9		Bloom, 2011 (61)	Peer reviewed	0.85
10		Bridges and Woolcock, 2017 (33)	Gray literature	0.99
11		Clement et al., 2015 (34)	Peer reviewed	0.84
12		Cole and McGinnis, 2017 (75)	Gray literature	0.88
13		De Bruyn, 2019 (35)	Peer reviewed	1.00
14		Desai and Snavely, 2007 (47)	Peer reviewed	0.97
15		Edelstein et al., 2018 (70)	Peer reviewed	0.95
16		Eriksen, 2007 (48)	Peer reviewed	0.93
17		Gagnon and Seguin, 2010 (57)	Peer reviewed	0.92
18		González-Block, 2009 (65)	Peer reviewed	0.85
19		Hudalah et al., 2017 (63)	Peer reviewed	0.88
20		Ihekweazu et al., 2015 (37)	Peer reviewed	0.88
21		Jakab et al., 2021 (14)	Peer reviewed	0.85
22		Johnstone et al., 2019 (38)	Peer reviewed	0.88
23		Rao and Khandelwal, 2016 (60)	Gray literature	0.85
24		Khan et al., 2020 (39)	Peer reviewed	0.76
25		Lejano, 2006 (71)	Peer reviewed	0.89
26		Lejars, 2008 (59)	Peer reviewed	0.85
27		Lenz, 2021 (49)	Peer reviewed	0.88
28		Leovaridis and Popescu, 2015 (50)	Peer reviewed	0.79
29		Lopert et al., 2017 (51)	Gray literature	0.86
30		Mahoui and Ferfera, 2013 (72)	Peer reviewed	0.79
31		Meda et al., 2016 (42)	Peer reviewed	0.45
32		Munteanu and Newcomer, 2020 (81)	Peer reviewed	0.88
33		Naimoli and Saxena, 2018 (73)	Peer reviewed	0.92
34		OECD, 2014 (67)	Gray literature	0.84
35		OECD, 2020a (76)	Gray literature	0.86
36		OECD, 2020b (85)	Gray literature	0.84
37		Pelling et al., 2008 (52)	Peer reviewed	0.81
38		Radin, 2020 (9)	Gray literature	0.99
39		Reinold, 2017 (68)	Gray literature	0.88
40		Romanelli, 2017 (82)	Peer reviewed	0.83
41		Rosenfeld et al., 2020 (43)	Peer reviewed	0.88
42		Ruseva et al., 2015 (53)	Peer reviewed	0.85
43		Savedoff, 2011 (77)	Gray literature	0.99
44		Treviño, 2016 (69)	Gray literature	0.85
45		Verrecchia et al., 2019 (74)	Peer reviewed	0.77
46		World Bank, 2014 (78)	Gray literature	0.86
47		World Bank and Government of Rwanda, 2020 (44)	Gray literature	0.75
48	MMAT	Abdullahi et al., 2016 (79)	Peer reviewed	1.00
49		Boyd, 2011 (56)	Peer reviewed	1.00
50		Buntaine, et al., 2017 (84)	Peer reviewed	1.00
51		Erondu et al., 2021 (36)	Peer reviewed	1.00
52		Essink et al., 2020 (62)	Peer reviewed	1.00
53		Grajzl and Murrell, 2009 (86)	Peer reviewed	1.00
54		Marjanovic, et al., 2012 (41)	Peer reviewed	1.00
55		Mayer et al., 2019 (58)	Peer reviewed	1.00
56		Portes and Smith, 2010 (10)	Peer reviewed	1.00
57		Salkic, 2014 (54)	Peer reviewed	1.00
58		Taytak and Aydin, 2019 (55)	Peer reviewed	1.00
59		Strielkowski et al., 2020 (80)	Peer reviewed	1.00
60		Song, 2009 (64)	Peer reviewed	1.00
61		Zaato and Ohemeng, 2015 (45)	Peer reviewed	0.90
62	NHLBI-NIH	Manoj et al., 2020 (40)	Peer reviewed	0.71

### 3.3. Domains of institution building

Using the “Framework Analysis” (30), we identified key domains of institution building and classified our results accordingly. All these domains encompass relevance to the process of establishing and developing NPHIs: (1) Governance; (2) Knowledge and innovation; (3) Inter-institutional cooperation; (4) Monitoring and control; (5) Participation; and (6) Sustainability and context-specific adaptability. In Table 5, we present the different documents selected considering the information according to the identified domains and our outcomes of interest: theories and concepts; framework and guidelines; and documents providing empirical evidence (e.g., case studies, evaluations, cross-sectional studies). The full information extracted from documents is presented in an extra table as Supplementary information.

**Table 5:** Classification by institution building domains and outcomes of interest.

		Outcome of interest			Theories and concepts	Frameworks and guidelines	Empirical evidence and modelling
Domains	Governance	Desai and Snavely (2007) (47) Edelstein et al. (2018) (70) Eriksen (2007) (48) Jakab et al. (2021) (14) Lejano (2006) (71) Mahoui and Ferfera (2013) (72) Rao and Khandelwal (2016) (60) World Bank and Government of Rwanda (2020) (44)	Abdellatif et al. (2019) (66) Abdullahi et al. (2016) (79) African Union and Africa CDC (2019) (22) Aluttis et al. (2014) (46) Bloom and Wolcott (2013) (83) Buntaine et al. (2017) (84) Clement et al. (2015) (34) Cole and McGinnis (2017) (75) Lopert et al. (2017) (51) Manoj et al. (2020) (40) OECD (2020a) (76) Pelling et al. (2008) (52) Rosenfeld et al. (2020) (43) Ruseva et al. (2015) (53) Savedoff (2011) (77) World Bank (2014) (78) Zaato and Ohemeng (2015) (45)	Alam et al. (2016) (31) Barma et al. (2014) (5) Binder et al. (2008) (23) Bloom (2011) (61) Bridges and Woolcock (2019) (33) Boyd (2011) (56) Gonzalez-Bock (2009) (65) Lejars (2008) (59) Marjanovic et al. (2013) (41) Mayer et al. (2019) (58) Salkic (2014) (54) Song (2009) (64) Portes and Smith (2019) (10)
	Knowledge and innovation	Desai and Snavely (2007) (47) Edelstein et al. (2018) (70) Leovaridis and Popescu (2015) (50) Mahoui and Ferfera (2013) (72) Naimoli and Saxena (2018) (73) World Bank and Government of Rwanda (2020) (44)	Abdullahi et al. (2016) (79) African Union and Africa CDC (2019) (22) Aluttis et al. (2014) (46) Bloom and Wolcott (2013) (83) Meda et al. (2016) (42) Munteanu and Newcomer (2020) (81) OECD (2020a) (76) Romanelli (2017) (82) Ruseva et al. (2015) (53) Zaato and Ohemeng (2015) (45)	Alam et al. (2016) (31) Binder et al. (2008) (23) Boyd (2011) (56) Erondu et al. (2021) (36) Essink et al. (2020) (62) Ihekweazu et al. (2015) (37) Gonzalez-Bock (2009) (65) OECD (2020b) (85) Portes and Smith (2019) (10)
	Inter-institutional cooperation	Desai and Snavely (2007) (47) Eriksen (2007) (48) Jakab et al. (2021) (14) Lejano (2006) (71) OECD (2014) (67) Reinold (2017) (68) Treviño (2016) (69) Verrecchia et al. (2019) (74) World Bank and Government of Rwanda (2020) (44)	Abdullahi et al. (2016) (79) African Union and Africa CDC (2019) (22) Aluttis et al. (2014) (46) Buntaine et al. (2017) (84) Bloom and Wolcott (2013) (83) Lenz (2021) (49) Manoj et al. (2020) (40) Meda et al. (2016) (42) Ruseva et al. (2015) (53)	Alam et al. (2016) (31) Barma et al. (2014) (5) Bloom (2011) (61) Bridges and Woolcock (2019) (33) De Bruyn (2019) (35) Grajzl and Murrel (2009) (86) Hudalah et al. (2017) (63) Ihekweazu et al. (2015) (37) Johnstone et al. (2019) (38) Lejars (2008) (59) Marjanovic et al. (2013) (41) Mayer et al. (2019) (58) OECD (2020b) (85) Portes and Smith (2019) (10) Radin (2020) (9)
	Monitoring and control	Desai and Snavely (2007) (47) Khan et al. (2020) (39) Naimoli and Saxena (2018) (73) OECD (2014) (67) Treviño (2016) (69) Rao and Khandelwal (2016) (60) World Bank and Government of Rwanda (2020) (44)	Abdellatif et al. (2019) (66) Abdullahi et al. (2016) (79) African Union and Africa CDC (2019) (22) Lopert et al. (2017) (51) Manoj et al. (2020) (40) OECD (2020a) (76) Romanelli (2017) (82) Taytak and Aydin (2020) (55) World Bank (2014) (78) Zaato and Ohemeng (2015) (45)	Alam et al. (2016) (31) Barma et al. (2014) (5) Binder et al. (2008) (23) Erondu et al. (2021) (36) Essink et al. (2020) (62) Marjanovic et al. (2013) (41)
	Participation	Desai and Snavely (2007) (47) OECD (2014) (67) World Bank and Government of Rwanda (2020) (44)	Abdullahi et al. (2016) (79) African Union and Africa CDC (2019) (22) Manoj et al. (2020) (40) OECD (2020a) (76) Romanelli (2017) (82)	Barma et al. (2014) (5) Binder et al. (2008) (23) Boyd (2011) (56) Essink et al. (2020) (62) Grajzl and Murrel (2009) (86) Marjanovic et al. (2013) (41) Mayer et al. (2019) (58)
	Sustainability and context specific adaptability	Desai and Snavely (2007) (47) Eriksen (2007) (48) Jakab et al. (2021) (14) Mahoui and Ferfera (2013) (72) Naimoli and Saxena (2018) (73)	African Union and Africa CDC (2019) (22) Aluttis et al. (2014) (46) Bloom and Wolcott (2013) (83) Buntaine et al. (2017) (84) Manoj et al. (2020) (40) Ruseva et al. (53) Strielkowski et al. (2020) (80)	Barma et al. (2014) (5) Bloom (2011) (61) Bridges and Woolcock (2019) (33) Boyd (2011) (56) Gagnon and Seguin (2010) (57) Hudalah et al. (2017) (63) Song (64)

#### 3.3.1. Domain 1: governance

The majority of the 62 documents described elements related to governance including sub-topics like ownership, strategy, leadership, and team building. Savedoff (77), in a policy report for the World Bank, states that the concept of “governance” has increasingly gained importance in the health sector when searching for strategies to improve the delivery of health care services. Rather than looking at inputs and outputs, questions about governance aim to identify factors that influence the behavior of the system, such as rules or procedures that might result in better performance and outcomes. The author further suggests that governance in the health sector can and should be measured, to help stakeholders in the decision-making process, proposing a set of indicators for both *governance performance*, that measures the gap between expected and actual behaviors, and *determinants of governance*, that are more complex and address policy instruments (e.g., existence of national health strategies or policies).

Political leadership was stated as “essential” to achieve successful reforms and the building of institutions (47, 48, 56, 78), including the enforcement of related regulations (61). According to lessons learned in the creation of NPHIs, leaders should have a clear vision, the ability to support staff, use opportunities, deal with controversies (23), and be committed to accomplish both political and technical objectives (53). Especially in the *health sector*, institutions are not only based on formal structures or on classic “bureaucratic modes of governance,” but strongly on relationships between policy actors and complex social systems (71). Considering NPHI, countries follow different organization and governance models, with strong difference in autonomy to Ministries of Health or other governmental organizations and different legal frameworks (43).

A clear vision and values of institutions are the basis for the work of the leaders, managers, and staff for achieving a good performance in the health sector (46), including NPHIs (22), and in other public sectors (45, 54). The OECD describes in its “Policy Framework on Sound Public Governance” (76) the values that should be considered: “integrity, openness, inclusiveness, and accountability.” Portes and Smith (10) demonstrated that one of the determinants of an institution of good quality is the avoidance of a “self-seeking union of bureaucracies” or “island of power,” meaning, an institution should avoid to rule for its own end, and, instead, should focus on its function. Empirical evidence of a study carried out in four “challenging contexts” (The Gambia, Lao People’s Democratic Republic, Sierra Leone, and Timor-Leste), shows that institutions can be strengthened even in fragile settings when leaders and managers use a set of tools: (1) making strategic choices, (2) adaptation of administrative architecture, (3) management of people (senior and new employees), (4) building of organizational identity, (5) leadership reaching beyond individuals, and (6) learning and self-evaluation (5).

##### 3.3.1.1. Empowered and effective leadership

Organizations should strengthen and promote effective leadership at all levels (22, 45, 84), enabling “innovative, coordinate, capable bureaucracy and (local) governments” (44). The establishment of an autonomous organization, with local ownership, not driven from external funders (40) and with independence in management and protected from political influence, is an important strategy to avoid misuse of power and partisan interference (5, 10, 45). Empowered leadership (40) and the appointment of a board of directors (45) are important factors in institution building, as well as the creation of “safe spaces” for mediation and exchange of ideas between stakeholders in a “non-accusatory manner” (58). In one publication on strengthening research institutes in Africa, the authors described the importance of senior actors (university vice-chancellors, deans, and senior researchers) leadership in lobbying for policymakers` commitment to evidence-based policy and resource mobilization (41). Besides, leaders and managers may strive for pragmatic solutions and “quick wins,” especially in settings of weak governance, to reinforce political support and to create virtuous cycles of change (31). With reference to “New Public Management,” Abdellatif et al. (66) propose leaders and managers to focus on improving efficiency and effectiveness (maximization of outputs), and to pay attention to budget procedures and to specific insights of public sector financial performance.

##### 3.3.1.2. Strategy, frameworks, and guidelines

Plausible and well-grounded strategic planning is another crucial aspect that institutions and organizations should focus on and implement (22, 32, 54, 59, 64, 73, 76, 84). Institutions should be built by design, not by chance (75). According to Clement et al. (34), strategic planning for institutional reform should cover the identification of the requirements, the development of a map picturing all the possibilities, and selecting most suitable options. With respect to reforms and institutional change, they should aim at “solving problems rather than selling solutions” (33). Considering some of the frameworks and guidelines related to governance and leadership, Pelling et al. (52), in the context of climate change adaptation of organizations, suggest six practical “adaptive pathways” usually taken for rethinking working routines, of which the most significant for institution adaptation sustainability being the “agent centered reflexive adaptation” (learning from experience to adapt goals and methods) and “agent centered institutional modification” (changes in the institutional context for future adaptive capacity and action – e.g. “scientific lobbies to change policy priorities”). Lopert et al. (51) describe a set of activities regarding institution building for “Health Technology Assessment” as follows: “(1) completing description of organizational structure, roles and responsibilities; (2) specify high level governance arrangements and regulatory reforms; (3) establish key partnerships with stakeholders; (4) identifying and addressing technical capacity needs; (5) identifying critical information and evidence (data gaps); (6) establishing relevant advisory committees and associated secretariats; (7) developing and promulgating guidelines with consultation feedback; (8) developing communication and stakeholder engagement strategies; (9) defining decision-making framework and process for policies.” The World Bank published a tool-kit for corporate governance of state-owned enterprises (SOE) (78) that describes different components for a SOE reform framework: (1) legal and regulatory framework, (2) state ownership arrangements, (3) performance monitoring, (4) financial and fiscal discipline, (5) professionalization of a board of directors, (6) transparency, disclosure and controls, (7) protecting shareholder rights in missed-ownership companies; and (8) implementation of reforms. The document also describes basic steps to be followed to develop a state-owned enterprise code, and strategies to implement reforms – securing political leadership and commitment, phasing, and sequencing reforms, gathering, and publishing data, supporting improvements, building institutional capacity, building support for reform among stakeholders and the public.

*Team building* and the generation of a good working environment inside an organization are important components of institution building (56, 60). Leaders and managers should identify and address staff needs, providing feedback for the accomplishments and tangible changes achieved in the institution building processes (60). Yet, cultural aspects and local management settings should be taken into account: even if many authors describe that the introduction of “team-based” management and optimization of processes may improve the governance and management of an institution (31, 54), Song (64) shows that such strategy may not be effective in a historical hierarchical administration setting. Leaders can encounter challenges to convince staff that changes are possible and can be positive, and that “new autonomies” for staff due to decentralization process demand higher accountability of results at lower levels – yet, these processes can be facilitated (79).

##### 3.3.1.3. Employees and code of conduct

Some of the documents reviewed refer to codes of conduct for public institutions and employees (58, 76). Methods like provision of incentives for the employees and decentralization of the governance of the institution were described as successful in South Africa (31). These codes can be “voluntary (not forcing), comply-or-explain codes and mandatory codes.” They should be widely disseminated, and stakeholders should receive training about their content. Compliance with such codes should be monitored, evaluated, and be part of the performance assessment processes (66).

#### 3.3.2. Domain 2: knowledge and innovation

One of the domains we identified in many of the documents reviewed is the importance of knowledge development, innovation, and technology production. Knowledge-based institutions and organizations place a high value on the employees, requiring motivating factors, proper and continuous training, and incentives to drive performance and assure sustainability (50, 60, 64, 74).

##### 3.3.2.1. Learning organizations

Theories, frameworks and empirical evidence (46, 56, 76) refer to the importance of knowledge development based on (health) information, reporting and research. For example, merely by developing a research agenda, the Ministry of Health of Laos improved its institutional capacity in terms of prioritizing the research needs of stakeholders (62). Similarly, institutional capacities are likely to increase if NPHIs pursue their core function: generating data and using evidence to elaborate recommendations and to feed into policy development. According to the African Union and the Africa Centres for Disease Control (Africa CDC), research priorities are often determined by donor interests and the availability of funds, leaving many critical questions unanswered (22). Hence, NPHIs should build up their capacities to define the research needed to answer questions critical to improving the country’s public health (22). Collaborative learning approaches can be implemented considering global lessons, but focusing on a regional or local learning effect (53). One strategy is to become a “learning organization” as described by Naimoli and Saxena (73), who suggest that ministries of health in low and middle income-countries may use the “3 M Framework” (“Meaning, Management, and Measurement” described by Garvin (87) in 1993), a framework that considers that ideas (“meaning”), explicit policies (“management”), and tools to assess systemic change (“measurement”) are essential to strengthen the learning capacities of an organization and to offer ideas to foster and institutionalize continuous learning. They describe that learning should not occur by chance, but by design. It should be centred on systematic problem-solving and be based on scientific methods and data. Besides, Munteanu and Newcomer (81) illustrate the importance to support institutions in providing relevant insights from data to formulate strategies and manage both the demand and supply of evidence. Yet, the use of pragmatic, flexible and innovative approaches is described as successful practice to make institutional reforms happen (31).

##### 3.3.2.2. Human resources

Knowledge-based organizations are dependent on highly-skilled human resources to be able to produce knowledge (50), and institution strengthening in the public sector depends on public-services employees. Five NPHI directors referred to the importance of well-trained personnel (23), supporting the importance of raising technical and managerial skills of human resources (45, 46, 78). Institutions and their personnel should learn from past experiences, peers and clients (47). Institutions may choose to include the development and management of knowledge to improve organizational performance, focus on both organizational performance and benefit of employees, or even have a primary focus on employees’ support that would bring consequent benefits for the organization. Still, it is important to consider motivational factors for employees, both financial and non-financial, to secure their loyalty (50).

##### 3.3.2.3. Innovative technologies

Technology may “drive and support the public sector management reforms” and its use helps public organizations to “rediscover a knowledge-based approach” that can strengthen their institutions (82). Innovations are determinants of good service provision of an institution (31, 56), and can as well contribute to increased transparency and, consequently, to the reduction of fraud and corruption. The use of new technologies (digitalization) enables institutions to “implement governmental information reforms more efficiently and effectively” (79) and simplifies the interface between citizens and the state (31).

##### 3.3.2.4. Support to the production of knowledge

The transfer of knowledge throughout the organization (73), including collection and data of local sources (36), should occur in a quick and efficient way. Stakeholders should be informed about the production of information and knowledge, and its benefits (83). Innovation is more likely to be successful if “central and local governments share a political commitment to create a supportive enabling environment” (44). Decision-makers may support research at different levels of intervention if they are interested in obtaining “critical feed-back on their policies and programs,” as demonstrated in a case study in Mexico on institution building and health system research (65).

#### 3.3.3. Domain 3: inter-institutional cooperation

Nearly half of the sources of evidence covered information related to cooperation between institutions at the national or international level. This can be condensed into three main sections:

##### 3.3.3.1. Like-minded organizations

Cooperation between like-minded organizations, organization that have similar inter-organizational and intersectoral aims and processes, is a good strategy for institutional change (37, 68). This includes sharing of resources, expertise, behavioral norms, core ethical values, and effective governance influencing health system performance (42, 83). Especially in the case of NPHIs and global health, data sharing and capacity building support between organizations is a critical element for the preparedness against global health threats (14, 38, 39). The data sharing process increases not only the capacity for disease detection but also the managerial capacities of the institutions involved in the process (70). Collaboratives of stakeholders can be established to analyze issues and generate knowledge (58, 63) that can be used for the elaboration and implementation of plans and strategies. Still, the lack of coordination between ministries and public sector agencies may come from unclear responsibilities of central and local authorities (44). To improve such relationships, continues interaction and close communication (41) and integrative systems (71) are necessary. External engagement with high-level institutional authorities and experts like academics, policymakers, healthcare practitioners, NGOs, among others are very important to strengthen capacity and generate impact (41).

##### 3.3.3.2. International cooperation

The engagement with international organizations as well as with other stakeholders like academics, policymakers, healthcare practitioners, NGOs “can help build the capacity of healthcare organizations, policymakers and the public to absorb and act on evidence and innovations” (41). The analysis of international experiences can be used as evidence to address domestical challenges (31). The OECD proposes that assistance and transfer of expertise are key strategies to provide support for institutional reforms (67). Different authors suggest key partnerships between institutions in transformation processes and (health) institutions with stronger capacities. The latter ones can bring in their expertise to the delivering on the Essential Public Health Functions (EPHFs). For example, they may support with capacity building activities, technology transfer, and provide structured peer-to-peer assistance that can be transformed into tailored and organized collaboration (14, 42, 47, 49, 67, 74).

Different authors described that many institutions and countries need financial support from international organizations (5, 9, 10, 22, 37, 38, 49, 59, 84, 85), especially in the areas of institutional change and reform management, human resources management, health promotion, performance measurement, staff recruiting and training (38, 59, 84). National policymakers and elites may encourage international donor support due to the need of financial resources, stimulating reforms and improving services and management systems (5); yet the inflow of international funds may inadvertently encourage opportunistic behaviors (84). Related to funding for research, some authors recommend the establishment of long-term capacity building in research instead of searching for grant-writing (22, 37, 85). In general, external funding and initiatives must respond to core domestic interests and local context, setting targets and establish processes that are appropriate to the aid-receptor characteristics (9, 41, 84), avoiding the simple transfer of “best-practices” that may work in one context, but not in another (33, 84). Foreign support is only beneficial if channeled selectively and adapted to the local context (35, 84, 86).

##### 3.3.3.3. Public-private partnerships

The private sector can be a source of financial resources for institution building in the public sector. Two publications describe the role of private funding or private investment in emerging economy institutions, highlighting the “interdependence between political institutions, capital markets, and infrastructure development” (69). González-Block (65) describes the private investment in research activities of the Mexican Health Foundation (FunSalud). This, in turn, has served to facilitate collaboration for policy development including academic organizations, government, private sector, other international institutions, and foundations. Yet, the authors also recognize limitations for the use of private funds in institution building, and that public institutions need support from the public sector.

#### 3.3.4. Domain 4: monitoring and control

Many sources of evidence in this review show that for any organization, measuring performance and results is crucial for institution building, and that there is a wide array of approaches and instruments ranging from performance measurement, monitoring and evaluation to different types of audits. As state-owned and public organizations often suffer from poor reputation, low legitimacy, and are often perceived by the public as being corrupt (45, 47, 66, 69, 79), control and the transparent dissemination of findings is critical.

##### 3.3.4.1. Measurement of performance and internal control

Continuous internal auditing and monitoring processes (36, 55, 74, 76) are good practice and indispensable for sound institutions. Still, staff should have adequate freedom do work independently, develop ideas and be empowered (60). Short-term measures are as crucial as medium- and long-term measures to demonstrate an organization’s level of learning (73). Yet, as Buntaine et al. (84) demonstrated, performance measurements based on international donors` agendas may fail; it is therefore important to note that such measurement systems need to be tailored to the context. Barma et al. (5) distinguish “three core sets of outcomes” for evaluating the success of NPHIs: measurable improvements and results, legitimacy of the performance with population involvement, and resiliency and durable institutions, enhancing impact over time. Also, engagement with stakeholders can contribute to improved quality of monitoring (23).

##### 3.3.4.2. External frameworks and evaluation

External evaluation committees and commitment to international agreements as, respectively, the Joint External Evaluation (JEE) or the International Health Regulations (IHR) provide a robust structure for measuring the preparedness of institutions including knowledge sharing during a public health emergency (39, 67). External supervision and control mechanisms like Supreme Audit Institutions (SAIs) (76) are important elements which, through enforcement of financial or technical rigor, contribute to strengthening institutions. External evaluation committees should have legal independence, full assessment capacity, and critical appraisal, assuring complete confidentiality and preventing conflict of interests. Yet, they should be regulated by high level authorities (e.g., the Ministry of Health) for their establishment and functioning, including areas of expertise, experience, roles, responsibilities, skills and remunerations (51).

##### 3.3.4.3. Transparency

Transparent management of resources is good business practice (22, 66, 79), increasing the recognition by the society and increasing its support (5, 44). The integrity of public sector institutions is a crucial basis to prevent corruption. This includes a close monitoring of the budget’s execution and spending, and a proactive dissemination of data and information (76), as well as the measurements of performance and processes along with the communication of the results to the public (31, 66, 85). Awareness campaigns and educational behavioral programs for all citizens, focusing on children and youth, is a way to seed a culture of engagement and integration with the public sector (76), thus strengthening social accountability. Last but not least, the use of technology can be powerful for improving such transparency processes and the engagement of citizens (43, 62), which leads to the next domain.

#### 3.3.5. Domain 5: participation

Participation should be well balanced, as policies and policymakers may empower some specific groups, but also marginalise others during the process (67). The benefit of collaboration and development and exchange of knowledge should outweigh the costs of participation (41). The only mathematical modelling paper included in this review suggests that the effects of civil society participation in the institution building process would not always be positive: in case interest groups are aligned and/or the intervention occurs in a fragile environment, participation could have even an adverse effect (86). “Particularly large networks bringing together partners with different levels of (research) capacity” are delicate to manage and can bring conflicts (41). For effective participation, stakeholders should be informed of regulations, rights and obligations, and receive guidance through the process (67).

NPHIs should engage in dialogue with key stakeholders to identify and prioritize gaps, proposing ways to address them, and elaborate immediate and longer-term action plans, monitoring their processes from the beginning (23).

Still, the involvement with clients, users, and other actors is a stronger predictor of institutional quality (5, 10, 31, 44, 56, 66). Use of staff experience or local (health) priorities to elaborate plans and strategies ensures “consensus-building,” ownership, adequacy and sustainability of organizations and institutions (40, 58). For example, the engagement of partners ensured that decisions of policymakers in specific research topics focused on main public needs, strengthening the capacities of the involved institution, the Ministry of Health (62). The African Union and Africa CDC consider in their “Framework for Development of National Public Health Institutes in Africa” (22) that “advocacy, communication and social mobilization … are approaches to engaging civil society in helping NPHI achieve its goals,” and that coalitions with civil society and private partners should be fostered.

#### 3.3.6. Domain 6: sustainability and context-specific adaptability

The success of an institution can be measured based on the population’s recognition, result’s improvement, and result’s and organization’s sustainability (5). A resilient institution has lasting results overtime, even in case of personal or leadership changes, or crises. For sustainability and resilient institutions, authors described the importance of the structure and design of the institutions (41, 69, 80). Hiring staff, building and renovating physical spaces need to have long-term funding and planning (72). Aspects as the ownership by governments, support of partners, and development of capacities to obtain funding is extremely important for NPHIs (22). For sustainability and adaptability, actors should consider context-specific aspects such as: public health institutional capacity, program and service-delivery structures, preparedness and capacity for emergency response, and financial aspects as resource generation and allocation (46). Public administration is affected by administrative and cultural traditions, and these should be strongly considered in the institution building processes (33, 47, 48, 56, 64, 84).

Organizations can be sustainable and resilient by making continuous adjustments and keeping flexible approaches, strengthening civil service capacity, and considering their strategy and mission (56, 57). Crises are also chances to expose debilities and change approaches (14). Ruseva et al. (53) proposed to drive the institutions “from elitism to populism, from centralism to decentralization, from isolated professionalism to dialogue and from percolation to growth.”

## 4. Discussion

This systematic review fills a major gap of aggregated information on institution building in the field of public health and National Public Health Institutes, and the need of synthesizing and summarizing the evidence including clear elements on how to tackle related challenges. It is to our knowledge the first systematic review of this kind. The overriding result is the identification and definition of six domains of institution building in the health sector: “governance,” “knowledge and innovation,” “inter-institutional cooperation,” “monitoring and control,” and “sustainability and context-specific adaptability.” The domains have been synthesized based on the most prominent recurrent themes and issues obtained from the 62 papers of the systematic review. In addition, the results show that health experts and managers are generally aware of the importance of these domains. Still, when it comes to institution building in the health sector, the concepts are not yet fully present in the discussions and not consistently applied in practice. Most of the documents cover separate elements of institution building using a variety of terminologies and concepts like e.g.: governance, strengthening of the health sector, support of National Public Health Institutes. Especially in articles related to the health sector, terms such as “strengthening” or “support” of organizations were frequently used, and there are documents that present holistic approaches to improve institutions, but without specifically using the term “institution building” (88). A system-thinking approach, considering different components of institution building and their interrelations is not fully recognized or described by most of the documents reviewed.

Most documents refer to the relevance of governance and leadership or monitoring and evaluation. Yet, there are few concrete or operative examples on how to improve governance of institutions, especially in the health sector. Recommendations often refer to common strategies such as that organizations should have clear functions and strategic plans. Few provide a comprehensive overview or present a coherent practical guidance, like in the example of the “toolkit for corporate governance of state-owned enterprises” developed by the World Bank (78), though not specifically for the health sector. The application of *knowledge and innovation* has been found to be critical for institution building. Yet, not only the application but also the production of information and its sharing needs to be fomented to advance the effects. Institutions and stakeholders should develop a continuous learning culture, with explicit policies and tools to institutionalize learning processes and provide space to develop ideas and design strategies (73). Moreover, knowledge and innovation need to go hand in hand. A recurrent recommendation of many authors is to increase the use of digital innovations. For instance, NPHIs functions like surveillance can be performed and enhanced with the use of information and communication solutions. However, applying new technologies is not sufficient. Quality, relevance and timeliness of data and information shared between countries and regions through such systems is of highest importance. Authors also recommend using technology and participation of civil society for monitoring performance of internal processes and public institution control. Constant innovation utilizing technology for planning, implementation and evaluation is also a core recommendation for assuring resilient, sustainable institutions. In our review we can conclude that technology and innovation is the cross-cutting aspect of all domains of institution building and organization strengthening.

As described by Eleonor Ostrom in her Institutional Analysis and Development (IAD) Framework (89), institutions can be strengthened and built by design. Cole and McGinnis (75) conclude that “the ability to communicate, reason, contest, understand, and commit makes it possible to design institutions by reason and choice rather than be subject to accident, force, tragedies or dilemmas.” Therefore, as can be derived from our results, organizations should be constantly *learning and producing knowledge* throughout three main ways: (1) strengthening human resources capacity, (2) measuring and managing data, and (3) sharing information.

Many documents, especially those describing cooperation at an international level, highlighted the importance of tailoring strategies and actions to context-specific needs, culture, and historical aspects. These findings go hand in hand with the importance that Ostrom and other authors attach to the contextual factors when analyzing institutions (13, 89, 90). Failures of development cooperation often stem from an over-reliance on Western concepts when implementing institution building initiatives (91). A lack of alignment with the needs and perspectives of stakeholders involved, and with communities they aim to serve, may lead to organizations which are ineffective and, in the end, unsustainable. Institution building in public health – as elsewhere – must therefore be context-specific and follow a country-driven approach.

We faced some methodological limitations in this systematic review due to the wide nature of the topic. We followed the PRISMA guidelines for systematic reviews, which primarily focuses on the reporting of reviews evaluating the effects of bio-medical interventions, mainly by using quantitative data. In this study, we have applied the guidelines for a systematic review using qualitative data in the form of public management themes and domains. The processing of such data, deciding what and how to extract, aggregate, and synthesize the information requires expert judgement. In order to identify the main domains of institution building, and partly in order to cope with this challenge, we made use of a qualitative tool, “Framework Analysis.” Since we aimed to include all the documents compiling information on how to tackle challenges for building and strengthening public health institutions, we found that many of the existent literature for this matter is rated as “gray.” There were no quality appraisal tools specific for this kind of literature, very different from typical clinical interventional or observational studies more commonly used in systematic reviews. The strength of our study is that we included this gray literature in the systematic review, adapting different tools to assess their quality and to analyze the information according to the type of document and study design.

In sum, our review revealed that strengthening public health institutions is not entirely different from other public sectors. In all scenarios, stakeholders may benefit from observing the aspects we classified as our main domains: governance, knowledge and innovation, inter-institutional cooperation, monitoring and control, participation and sustainability and context specific adaptability. Here, it is important to consider both the specific aspects, or domains, of institution building as well as their interrelationship, using a system thinking approach. By doing so, it becomes possible to identify and address potential interrelated challenges, promote synergies, and enhance the overall effectiveness and sustainability of the institution building process. Such an approach can help to ensure that the institution building initiatives are better suited to complex and dynamic contexts, e.g., of specific countries, increasing their capacities to cooperate and coordinate at the international level, and consequently leading to better outcomes both locally and globally. The review results confirmed principles that have guided IANPHI in its peer-to-peer activity to support the development of NPHIs, as a process of advocacy and diplomacy vis à vis relevant stakeholders and decision-makers, taking a participatory approach, and adhering to country-ownership (17). The process should include a detailed situation mapping, identifying existing capacities and how they link with the entire system (45, 46). The review gathered many important sources of information and critical reflections on institution building beyond what can be reproduced in this paper.

One of the lessons learned during the COVID-19 pandemic was the importance of strong and agile NPHIs for resilient health systems (19). In our globalized world, NPHIs and international coordination between them are key to emergency preparedness and response. Therefore, national policy makers and global stakeholders should continue to support and strengthen the establishment and development of these institutions. Organizations and initiatives working on the creation, development, and support of public health institutions may benefit from considering the findings from this systematic review. The documents processed and referenced in the study form an additional source for further reading and input for the revision of the IANPHI framework.

## Data availability statement

The original contributions presented in the study are included in the article/Supplementary material. The Supplementary material comprises a spreadsheet document with the quality appraisal of the literature reviewed (Supplementary material 1), a framework analysis table with the key information of the literature reviewed (Supplementary material 2), and a table with the excluded documents and the reasons of exclusion (Supplementary material 3). Further inquiries can be directed to the corresponding author.

## Author contributions

LB developed the study protocol, data analysis, and wrote the first draft manuscript. LB and CJ carried out the searches, title and abstract review, full text review, data extraction, and quality appraisal. CJ also supported in the data analysis and manuscript writing. MO and LK supported in the search procedures and in the extraction of data. MM gave technical advice in the thematic institution building and organization strengthening. AD, OH and PD supported in the methodology, the development and application of the search strategy, and the selection of the articles. AF developed the research question, oversaw the systematic review process, and supported in the final manuscript writing. All authors contributed to the article and approved the submitted version.

## Funding

This systematic review received financial support from Robert Koch Institute, Berlin, Germany.

## Conflict of interest

The authors declare that the research was conducted in the absence of any commercial or financial relationships that could be construed as a potential conflict of interest.

## Publisher’s note

All claims expressed in this article are solely those of the authors and do not necessarily represent those of their affiliated organizations, or those of the publisher, the editors and the reviewers. Any product that may be evaluated in this article, or claim that may be made by its manufacturer, is not guaranteed or endorsed by the publisher.
